# Twelve tips for adopting the virtual Nominal Group Technique (vNGT) in medical education research

**DOI:** 10.12688/mep.19603.1

**Published:** 2023-03-23

**Authors:** Faraz Khurshid, Elizabeth O’Connor, Rachel Thompson, Iman Hegazi

**Affiliations:** 1 School of Medicine, Medical Education Unit,, Western Sydney University, Sydney, NSW, 2560, Australia; 2 Institute for Interactive Media & Learning,, University of Technology Sydney, Sydney, NSW, 2007, Australia

**Keywords:** Virtual Nominal Group Technique (vNGT), Social Distancing, Video conferencing

## Abstract

Nominal Group Technique (NGT) is a structured approach to consensus development and data collection driven by problem-solving, idea inception and prioritisation. Challenges of the coronavirus disease 2019 (COVID-19) pandemic necessitated the development of a virtual (vNGT) model to recruit participants from diverse locations and time zones. Our reflections reveal the opportunities and challenges of using Zoom ^©^ for NGT sessions, resulting in more effective engagement and focus with fewer distractions compared to in-person meetings. The 12 tips provide practical suggestions for expanding the versatility of NGT in a virtual environment. These recommendations cover every aspect of the process, including the person, place, and object, from planning the sessions, and utilising technology resources effectively, to ensuring a seamless implementation to desirable outcomes. The paper strives to assist individuals in effectively using the online NGT as a substitute for in-person events, promoting effective management of remote participants even during unprecedented times of quarantine and physical distancing.

## Introduction

The coronavirus disease 2019 (COVID-19) pandemic has disrupted the status quo of higher education teaching and research over the past three years. It has significantly influenced the process of data collection and has required rapid transformation of conventional face-to-face models to socially distant methods ( Lobe *et al*., 2020). The availability of, and rapid improvement in, numerous digital-based virtual platforms has remodelled face-to-face components of education and qualitative educational research. One clear benefit of virtually conducted research includes the ease of recruitment and communication with potential participants, using inexpensive but practical software that can manage complex research arrangements, ensure research continuity and foster meaningful social collaboration among participants ( Beddows, 2008).

**Figure 1.**
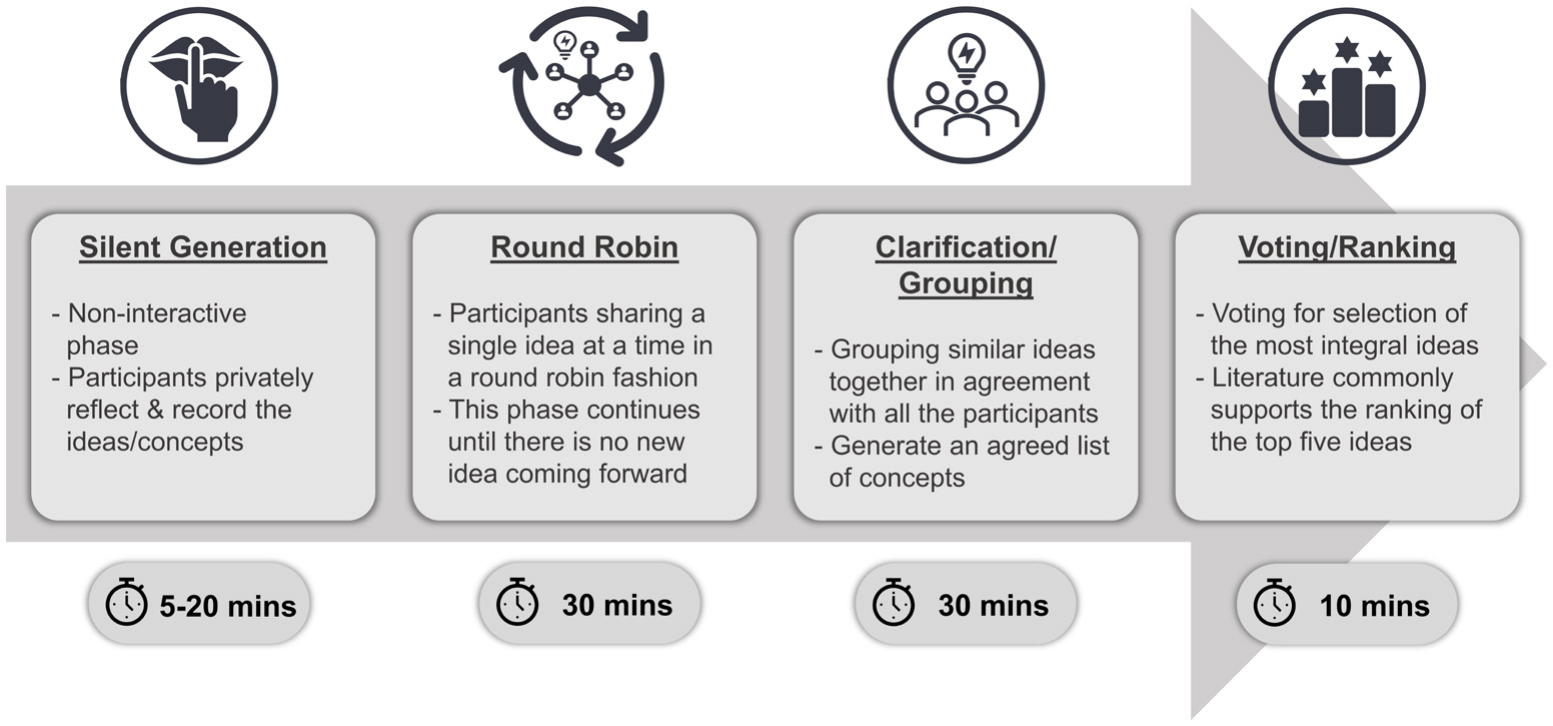
Schematic diagram of the classical steps of NGT. This figure is an original figure produced by the first author for this article.

Nominal Group technique (NGT) is an approach that traditionally runs as a face-to-face small group discussion, generating immediate data ( McMillan *et al*., 2016). It was envisioned that this process could be helpful in developing diverse perspectives in a structured manner, reducing the dominance of participants during the discussion, and increasing individual participation ( Delbecq *et al*., 1975; De Ruyter, 1996; Marques *et al*., 2021). NGT can be used as an alternative to focus groups when specific consensus-driven outcomes are sought. This consensus-obtaining method is devoted to problem-solving, notion development and establishing priorities. The problem is presented to the consented participant group as a nominal question, thus generating discussion around ideas by individual participants. Subsequently, a voting/ranking phase helps the participants to prioritise and agree upon the top ideas generated. These four steps of NGT are shown in the schematic diagram below ( Figure 1)

To modify NGT to suit the ‘social distancing’ and lockdown challenges of the COVID-19 pandemic, we adopted a virtual model of consensus generation, structured to ensure participant engagement. The virtual data collection also paved an alternative route to engaging time-challenged professionals. The transformation from in-person to online did not alter the original layout except the format of data collection. This paper shares 12 tips based on our experience with virtual NGT.

## Tip 1: Planning is the key

The COVID-19 pandemic has impacted qualitative research plans by disrupting normal activities and forcing researchers to alter data collection methods thus adapting to the changed circumstances created by the pandemic. Similarly, a thorough preparation and planning process is necessary for the implementation of NGT. This requires convening participants for face-to-face meetings, which can pose difficulties and result in low turnout ( Manera *et al*., 2019). A virtual focus or nominal group session should be planned based on several factors, including technology, participant demographics, optimal group sizes, ethical recruitment approaches, experienced moderators, favourable scheduling, clearly formulated research questions, and ethical considerations like obtaining informed consent. A participant's quality of experiences and knowledge is considered more important than quantity when ensuring data validity ( Potter *et al*., 2004).

Classical nominal groups generally have between six to eight participants ( McMillan *et al*., 2015). Given the availability of experts and students, our group sizes were effective, despite being three to five participants. Based on this, we recommend that a minimum of three and a maximum seven participants should be assembled in a virtual setting that is easily accessible for all participants, regardless of their geographical location, and at a time that is suitable for everyone. This method has a wider scope in medical education research by engaging health professionals and teachers who lack the resources to attend in-person sessions. In addition, virtual methods often ease the process of recruitment and participant engagement, minimising the burden of participants’ travel and logistics yielding a faster turnaround time ( Beddows, 2008; Rupert *et al*., 2017).

## Tip 2: Ease the process of recruitment

The pandemic limited opportunities for convenience sampling because in-person attendance was not available, so we recruited via purposeful sampling by email invitations. Purposive sampling can be applied, where participants with a diversity of demographic characteristics are selected to obtain a range of perspectives, since all eligible participants cannot be recruited ( Manera *et al*., 2019). Besides obtaining consent from all participants, an online scheduling poll should be conducted in order to determine a convenient and common session time for all participants.

Using a virtual recruitment platform can facilitate qualitative recruitment by leveraging social media, existing networks, partnerships, incentives, and easy-to-use tools while protecting participants' privacy and confidentiality. Choosing the right sampling method is crucial for a qualitative research study. For instance, purposive sampling was the appropriate fit for our research objectives and the consensus generation methodology we employed. By carefully selecting individuals deemed essential to the study, we were able to effectively engage participants and generate the desired consensus.

## Tip 3: Configure the groups and get started

Given that pandemic recruiting poses several challenges, it is vital that the process begins as soon as a minimum number of participants has been recruited. The composition of groups for exploratory purposes may be homogeneous if all participants have a similar level of experience, and heterogeneous if the group is composed of a diversity of participants (i.e., students and experts) ( Krueger, 2014; Muijeen *et al*., 2020). We initially began with separate ‘homogenous ‘sessions for experts and students to meet the minimum number of participants for NGT. As time moved on, the participants numbers increased and reached a maximum of five, bringing more heterogeneity to the process of data collection. Due to power differentials, participants in traditional NGT with less power may feel unable to express their own views or counter powerful participant's views ( McMillan *et al*., 2016). However, we found that the virtual adoption of NGT and its structured format did not result in a significant impact from power differentials as it allowed both high and low power participants ample opportunity to voice their opinions.

## Tip 4: Sensitize the participants using a pre-elicitation technique

Online data collection can be problematic if participants are not informed adequately about what they need to do beforehand or if inconsistencies are not addressed, leading to possible biases and decreased efficiency. Using the pre-elicitation technique, researchers can inform participants adequately and give them time to reflect on their views prior to the session ( Gonzales & Leroy, 2011; McMillan *et al*., 2015). With the virtual format of the NGT, this time-saving activity prepares participants better for the session, as some of the silent generation phase of the NGT can be completed prior to entering the session. As part of our research preparation, we created a matrix template that summarised our research query, along with guidelines for completing it. Participants used the matrix to note ideas or concepts related to threshold concepts in pharmacology. Additionally, we created a short video explaining the research focus and procedure to ensure smooth implementation. Additionally, this preparation tool provided participants with a chance to reflect on conceptual aspects they would encounter and their own experiences of this topic in relation to the research question, which sped up the process and prepared them better for the session.

## Tip 5: Harness videoconferencing technologies

Qualitative researchers face physical, psychological, and ethical obstacles during a pandemic since social distance and travel restrictions prevent in-person fieldwork ( Santana *et al*., 2021). Thus, virtual avenues have been adopted to collect data, such as virtual focus groups, which provide a 'promising alternative' to in-person focus groups and help overcome geographical barriers ( Marques *et al*., 2021). We chose Zoom ^©^ as the online videoconferencing software for gathering qualitative data as it is relatively easy to use and cost-effective, with useful data management features, security, and recording options ( Archibald *et al*., 2019). Further, our university recommends using a secure data system or maintaining the confidentiality of research data through the use of software programs such as Zoom ^©^ (or Microsoft ^®^ Teams) for videoconferencing. Additionally, real-time virtual software enables easy, high-quality audio-video recordings, which can be used to transcribe the meeting conversation and observe non-verbal behaviour to augment or complement data analysis ( Matthews *et al*., 2018). Automatic transcription speeds up the generation and analysis of data, improving timeliness and reducing the time and money spent on manual transcription ( Carter *et al*., 2021; McMullin, 2021; Singh *et al*., 2022). Despite occasional imperfections in the Zoom ^©^ auto-transcription, it was fairly comprehensible due to close review of the transcripts and consideration of the participants' non-verbal actions (via the video recordings) during data analysis.

## Tip 6: Harmonise the virtual generation of consensus

To ensure that the consensus generation session runs smoothly without major disruptions, a stable internet connection is vital. The beginning and end of the different steps of NGT can be signposted to the participants through the screen-share feature of videoconferencing platforms such as Zoom ^©^ or Microsoft ^®^ Teams. Similarly, during the round-robin phase, the facilitator can use the same feature to openly list and share the ideas proposed by individual participants. Our participants used the direct chat feature to communicate privately with the facilitator any doubts or misconceptions that they did not want to discuss openly. This feature also minimises the risk of openly discussing trivial issues that can often be distracting and irrelevant to the active research session. By harmonizing NGT with the virtual platform of Zoom ^©^, the structured format encourages participants to speak in turn. Therefore, everyone has the opportunity to make a contribution, instead of allowing the louder participants to determine the direction, thus resolving issues related to unequal power dynamics.

## Tip 7: Empower virtual teams through facilitation

Having a skilled facilitator/moderator is essential for ensuring the success of virtual team meetings and guiding the team towards their objectives ( Humphrey-Murto *et al*., 2017; Kayser, 2011). The facilitator encourages participation, guides the process and may prepare an ice-breaker to build rapport and create a comfortable environment for, and free-flowing discussions ( Manera *et al*., 2019). The facilitator, through their expert knowledge of group dynamics, plays a crucial role in creating an environment that promotes equal participation and open communication, allowing all members of the group to have their voices heard and their perspectives valued, resulting in a more effective and inclusive discussion.

The Nominal Group Technique is a time-effective method for collecting data as sessions typically last 1.5–2 hours with participants only needing to attend one session, but virtual interactions can be cognitively demanding as they require concentration of listening and viewing. ( Epstein, 2020; McMillan *et al*., 2016; Potter *et al*., 2004). As a result, the facilitator's proactive role in reducing this fatigue and preventing its dissemination is more valuable for effective consensus generation. NGT's structured stepwise, flexible format complements the role of the facilitator, who can either minimise or prolong the steps in accordance with the level of enthusiasm and motivation of the group in general. In the round-robin phase of NGT, for example, having a professional scribe can shorten the process and improve productivity. The scribe accurately records ideas, freeing the facilitator to focus on encouraging participation and maintaining the meeting flow by encouraging individual participants to share their thoughts. By streamlining, enhancing, and promoting this process, the round-robin phase will yield a more effective and efficient outcome.

## Tip 8: Reinforce ground rules and effective timekeeping for each phase

The virtual platform encourages one speaker at a time and can be effectively managed by reminding participants of the ground rules. It makes it easier to keep track of who proposes each idea or concept if there is both a facilitator and a scribe. The facilitator can keep track of the ideas and their originators with the help of a scribe's notes, available on a backup screen. For the virtual process, the ground rules should be set not only at the beginning but also at the inception of each NGT phase as verbal reminders or via a presentation slide that outlines the do's and don'ts of the process. Once this is accomplished, virtual sessions can generate succinct and relevant responses with minimal interruptions, side conversations or distractions than might be expected with in-person sessions.

The clarification/grouping phase is time-consuming, with completion time varying based on group size, number of questions, and type of participants ( McMillan *et al*., 2016). For example, a NGT for one question requires ~two hours, while a NGT for two questions could require one half-day, followed by another half-day for the forum event ( Bradley *et al*., 2013; Hutchings *et al*., 2010). Aside from keeping the virtual session focused on one nominal question, timekeeping is of paramount importance for a productive session, as it ensures fair allocation of presentation time and helps maintain focus and participant engagement by reducing the risk of boredom and fluctuations in concentration during extended periods ( Davis *et al*., 2021; Epping *et al*., 2020). For time-based tasks, Zoom Timer offers customizable, on-screen countdown solutions, with a maximum time limit of 100 minutes. For time-based tasks, Zoom Timer offers customizable, on-screen countdown solutions, with a maximum time limit of 100 minutes.

## Tip 9: Easy voting using online polls

In the private or confidential section of voting, participants may choose to prioritise all of the ideas ranked one through five, with the most significant ideas being ranked first, the next two ranked second etc., or to score each idea, such as assigning a number from one to five to each idea ( Manera *et al*., 2019; Vander Laenen, 2015). According to the literature, the nominal group technique typically involves ranking five concepts ( Delbecq *et al*., 1975; Dening *et al*., 2013; McMillan *et al*., 2015). Voting frequency is also calculated for the top five themes to gauge how often they were voted for and thus how popular they are. A vNGT session utilises the same overall timeframe for voting as classical NGT (~10 minutes). However, vNGT has the added benefit of being able to use a proprietary program to assist with this process, such as Qualtrics (Provo, UT) or SurveyMonkey (Momentive Inc., San Mateo, California, USA). In our study, the facilitator used Zoom ^©^ chat to share a secure online form link directly with each participant in our case, speeding up the whole process.

## Tip 10: Make the most of the flexibility of the virtual place

The inherent flexibility of the virtual format allows the vNGt to be customised to meet the needs of participants and researchers ( Manera *et al*., 2019). However, technology issues like internet blackouts, computer failures, and hardware malfunctions can pose challenges. Having reliable IT assistance and a backup plan in place can help rectify these unforeseen events. Moderators should also keep participants' contact information and establish clear agreements regarding their use to provide instant assistance in case of technological problems. Facilitators should have a backup plan in place in case the online platform fails ( Carter *et al*., 2021). This plan may involve switching to alternative technology, direct communication with participants, postponing the data collection, or conducting in-person data collection. By being prepared, the data collection process can continue smoothly, even in the event of technical difficulties. NGT's innovative approach ensures smooth session proceedings, even in the face of technology disruptions affecting some participants. Our scheduling system locks in two time slots for the same group, providing a backup option in case of any centralised interruptions.

## Tip 11: Wrap-up the experience

Holding a post-research evaluation or wrap-up session after a technology- or online-facilitated session is vital to make sure the technology and platform fulfil their purpose and participants have a positive and productive experience. Research methods can be improved by using participants' feedback not only to refine future designs and processes but also to provide support to methodological studies ( Carter *et al*., 2021). The wrap-up session not only gave us the chance to assess the outcome of the session and pinpoint areas for improvement but also provided valuable information about the technology and platform employed. Participants' feedback was instrumental in determining the technology's effectiveness and the impact of the online platform on their involvement and contribution.

## Tip 12: Immerse yourself in the data

Make use of the autogenerated audio-visual recordings to immerse yourself in the data. This is done by visiting or revisiting each interview recording to gain content immersion, generate field notes, and independently note their analytical interpretations. It is possible to take brief field notes, which may be limited to keywords, phrases, and thoughts, during a virtual session, but the audio-visual recording can provide a valuable backup tool for data analysis, which is not possible when conducting the session in real time, provided a recording system is available ( Santana *et al*., 2021). The audio-video recordings served as a valuable resource in our data analysis, utilizing an abductive line of reasoning. This approach allowed us to revisit the recordings, combined with data notes and transcripts, to reconstruct the experience and bring the observations to life in new and innovative ways.

## Major vNGT limitations

Note that we do not recommend vNGT for sensitive research as it may not be ideal for handling sensitive topics or engaging vulnerable groups of participants as it would be difficult to provide the appropriate individual support via this virtual arrangement. Although, it is possible to provide immediate and personalised assistance to a distressed participant by inviting them to a private breakout room if necessary, during the data collection process. Another major limitation to the virtual format is that participants in rural and indigenous communities may have significant access challenges to broadband internet, software or an appropriate device to join a meeting online.

## Conclusion

This unconventional execution of nominal group technique (NGT) in the pandemic era of COVID-19 helped us to run the process holistically without changing its essence and structure. The virtual format of NGT (vNGT) can serve as an effective alternative to the face-to-face method. It allows for engagement of participants placed in geographically distinct regions or time zones. In addition, virtual avenues for consensus generation do not adhere solely to the social distancing order but may also be used as comparable alternative to in-personal attendance in various educational, scientific and working environments where time is short or virtual attendance is advantageous. The 12 tips provided for the virtual adoption of nominal group techniques aim to guide and support the research process in a virtual setting. By effectively implementing these tips, you can ensure the successful and efficient adoption of this technique in a virtual environment, leading to meaningful and productive outcomes for your research.

## Ethics approval

We confirm that all methods used in the study were carried out in accordance with relevant guidelines and regulations. Any aspect of the work covered in this manuscript involving human subjects has been conducted with the ethical approval of the relevant bodies.].

## Data Availability

No data are associated with this article.

## References

[ref-1] Archibald MM Ambagtsheer RC Casey MG : Using Zoom Videoconferencing for Qualitative Data Collection: Perceptions and Experiences of Researchers and Participants. *Int J Qual Methods.* 2019;18:1–8. 10.1177/1609406919874596

[ref-2] Beddows E : The Methodological Issues Associated With Internet-Based Research. *Int J Emerg Technol Learn.* 2008;6(2):124–139. Reference Source

[ref-3] Bradley F Schafheutle EI Willis SC : Changes to supervision in community pharmacy: pharmacist and pharmacy support staff views. *Health Soc Care Community.* 2013;21(6):644–54. 10.1111/hsc.12053 23718766

[ref-4] Carter SM Shih P Williams J : Conducting qualitative research online: Challenges and solutions. *Patient.* 2021;14(6):711–718. 10.1007/s40271-021-00528-w 34114170 PMC8192219

[ref-5] Davis RK Chunta KS Gerwick M : Virtual meetings: reflecting on lessons learned from the past year to create a systematic approach to more effective meetings. *J Contin Educ Nurs.* 2021;52(9):423–428. 10.3928/00220124-20210804-08 34432576

[ref-6] De Ruyter K : Focus versus nominal group interviews: a comparative analysis. *Mark Intell Plan.* 1996;14(6):44–50. 10.1108/02634509610131153

[ref-7] Delbecq AL Van de Ven AH Gustafson DH : Group techniques for program planning: A guide to nominal group and Delphi processes.Glenview, USA: Scott, Foresman and Company,1975. Reference Source

[ref-8] Dening KH Jones L Sampson EL : Preferences for end-of-life care: A nominal group study of people with dementia and their family carers. *Palliat Med.* 2013;27(5):409–17. 10.1177/0269216312464094 23128905 PMC3652642

[ref-9] Epping E Lohse AP Graf L : Conferences and international collaboration revisited in times of the coronavirus: Experiences from a digital transition and lessons for the future. 2020. Reference Source

[ref-10] Epstein HAB : Virtual meeting fatigue. *J Hosp Librariansh.* 2020;20(4):356–360. 10.1080/15323269.2020.1819758

[ref-11] Gonzales CK Leroy G : Eliciting user requirements using appreciative inquiry. *Empir Softw Eng.* 2011;16(6):733–772. 10.1007/s10664-011-9156-x

[ref-12] Humphrey-Murto S Varpio L Gonsalves C : Using consensus group methods such as Delphi and Nominal Group in medical education research. *Med Teach.* 2017;39(1):14–19. 10.1080/0142159X.2017.1245856 27841062

[ref-13] Hutchings HA Rapport FL Wright S : Obtaining consensus regarding patient‐centred professionalism in community pharmacy: nominal group work activity with professionals, stakeholders and members of the public. *Int J Pharm Pract.* 2010;18(3):149–58. 20509348

[ref-14] Kayser TA : Building team power: how to unleash the collaborative genius of teams for increased engagement, productivity, and results.McGraw Hill Professional. 2011. Reference Source

[ref-15] Krueger RA : Focus groups: A practical guide for applied research. *Sage publications.* 2014. Reference Source

[ref-16] Lobe B Morgan D Hoffman KA : Qualitative Data Collection in an Era of Social Distancing. *Int J Qual Meth.* 2020;19:1–8. 10.1177/1609406920937875

[ref-17] Manera K Hanson CS Gutman T : Consensus methods: nominal group technique.In: Liamputtong P, editor. *Handbook of Research Methods in Health Social Sciences.* Singapore: Springer;2019;737–750. 10.1007/978-981-10-5251-4_100

[ref-18] Marques ICDS Theiss LM Johnson CY : Implementation of virtual focus groups for qualitative data collection in a global pandemic. *Am J Surg.* 2021;221(5):918–922. 10.1016/j.amjsurg.2020.10.009 33070983 PMC7550163

[ref-20] Matthews KL Baird M Duchesne G : Using Online Meeting Software to Facilitate Geographically Dispersed Focus Groups for Health Workforce Research. *Qual Health Res.* 2018;28(10):1621–1628. 10.1177/1049732318782167 29911490

[ref-21] McMillan SS Kelly F Sav A : Consumers and carers versus pharmacy staff: Do their priorities for Australian Pharmacy Services align? *Patient.* 2015;8(5):411–22. 10.1007/s40271-014-0105-9 25512020

[ref-22] McMillan SS King M Tully MP : How to use the nominal group and Delphi techniques. *Int J Clin Pharm.* 2016;38(3):655–62. 10.1007/s11096-016-0257-x 26846316 PMC4909789

[ref-23] McMullin C : Transcription and qualitative methods: Implications for third sector research. *Voluntas.* 2021;34:140–153. Reference Source 34522070 10.1007/s11266-021-00400-3 PMC8432276

[ref-24] Muijeen K Kongvattananon P Somprasert C : The key success factors in focus group discussions with the elderly for novice researchers: a review. *Journal of Health Research.* 2020;34(4):359–371. 10.1108/JHR-05-2019-0114

[ref-25] Potter M Gordon S Hamer P : The nominal group technique: a useful consensus methodology in physiotherapy research. *NZ Journal of Physiotherapy.* 2004;32(3):126–130. Reference Source

[ref-26] Rupert DJ Poehlman JA Hayes JJ : Virtual Versus In-Person Focus Groups: Comparison of Costs, Recruitment, and Participant Logistics. *J Med Internet Res.* 2017;19(3):e80. 10.2196/jmir.6980 28330832 PMC5382259

[ref-27] Santana FN Wagner CH Rubin NB : A path forward for qualitative research on sustainability in the COVID-19 pandemic. *Sustain Sci.* 2021;16(3):1061–1067. 10.1007/s11625-020-00894-8 33495701 PMC7816056

[ref-28] Singh H Tang T Thombs R : Methodological Insights From a Virtual, Team-Based Rapid Qualitative Method Applied to a Study of Providers’ Perspectives of the COVID-19 Pandemic Impact on Hospital-To-Home Transitions. *Int J Qual Methods.* 2022;21:16094069221107144. 10.1177/16094069221107144 35721871 PMC9189180

[ref-29] Vander Laenen F : Not just another focus group: making the case for the nominal group technique in criminology. *Crime science.* 2015;4(1):1–12. 10.1186/s40163-014-0016-z

