# Supplementary material for: Twelve tips for adopting the virtual Nominal Group Technique (vNGT) in medical education research
Source: MedEdPublish (2016). 2023 Mar 23;13:18. [Version 1] doi: 10.12688/mep.19603.1 (PMC10362375; doi:10.12688/mep.19603.1)
Supplement: Peer review history

# Peer review: Reviewer response for version 1

**DOI:** 10.21956/mep.21000.r33939

**Published:** 2023-08-01

**Authors:** Knut Fredriksen

**Affiliations:** 1 UiT The Arctic University of Norway, Tromsø, Norway

This paper describes practical tips for how to apply the nominal group technique with virtual platforms, particularly relevant during the Covid-19 pandemic, but even applicable in other settings. The practical tips may be of great help for other researchers to undertake vNGT studies and may help them to avoid existing pitfalls.

 I have some suggestions that may improve the manuscript:

1. I suggest a little bit more extensive presentation of the NGT: see e.g., the de Ruyters reference, and e.g., the Wikipedia discussion of NGT. These references present more possibilities to vary the NGT-based study design. There are many successful modifications of the technique that deserves to be mentioned. Furthermore:
  1. The optimal number of participants depends very much on the purpose of the study. It may often be much higher than suggested by the authors, especially to ensure a rich harvest of opinions (in terms of qualitative methods: saturation of themes) during the process. De Ruyters suggest ten participants. I would like to see a short discussion of this in the paper.
  2. It is unclear to me what “nominal” means. Is it the group that is nominal or the research question(s)?
  3. I suggest that the NGT is presented in a way that explains that it may be modified in several ways to fit the purpose of the study. This is reflected both by the basic references, but even more by searching for references that have applied the NGT for different purposes. In this respect the (otherwise nice) figure is too simple to explain what NGT can be.
2. I also would like to suggest a brief discussion of the purposive sampling of participants a and how choice of participants (together with number of participants) may affect the richness of, or undesirable bias in, themes that are generated for the later stages in the NGT process.
3. The time frames in NGT may differ, again depending on the purpose. What are the times referred to in the figure? This should be explained in the figure legend.  
4. The authors refer to their own application of virtual NGT without including it in the reference list. If it has been published it should be referred to, if not it should be explained better what it was about. This would help the reader to understand more of the methodological choices that are made by the authors.      

If evidence from practice is presented, are all the underlying source data available to ensure full reproducibility?

Not applicable

Is the topic of the practical tips discussed accurately in the context of the current literature

Partly

Are all factual statements correct and adequately supported by citations?

Partly

Are the conclusions drawn balanced and justified on the basis of the presented arguments?

Yes

Are arguments sufficiently supported by evidence from the published literature and/or the authors’ practice?

Partly

Reviewer Expertise:

Emergency medicine, clinical medicine, qaualitative research, medical education

I confirm that I have read this submission and believe that I have an appropriate level of expertise to confirm that it is of an acceptable scientific standard, however I have significant reservations, as outlined above.

# Peer review: Reviewer response for version 1

**DOI:** 10.21956/mep.21000.r33093

**Published:** 2023-07-21

**Authors:** Janneke Frambach

**Affiliations:** 1 Department of Educational Development and Research, School of Health Professions Education (SHE), Maastricht University, Maastricht, The Netherlands

The paper is written in a clear way and the twelve tips are relevant for those considering to apply vNGT. I feel the paper adds to the literature on Nominal Group Technique because it specifically focuses on using this in a virtual environment. This is especially relevant because we increasingly use virtual environments for data collection. I think the authors succeeded well in focusing on those aspects that are different for a virtual setting compared with a face to face setting. 

 Here are some further thoughts that the authors may consider:

 The authors mentioned vNGT under the umbrella of qualitative research, but in my view it is not a fully qualitative approach, for example considering the ranking involved. Perhaps the authors could consider using the umbrella of consensus group methods as an alternative?

 The authors’ explanation of purposive sampling (in Tip 2) does not fully resonate with my understanding of purposive sampling. I think purposive sampling is not necessarily about diversity but about sampling according to certain deliberately chosen criteria (which often could be about including a diverse sample, e.g. maximal variation sampling, but not necessarily).

 In tip 2 (ease the process of recruitment) the authors recommend the use of virtual recruitment platforms, which I think could also happen with face-to-face methods, and I would say there is convenience sampling involved in it as well.

 Tip 11 about wrap up is quite general, perhaps the authors could be slightly more concrete about the type of questions asked at participants? And does it mean you would have to schedule two sessions with the participants? Why not simply do this right after the NGT session? Perhaps the authors can clarify this further.

 Perhaps I missed it, but I did not see a reference of the authors’ own work on which they based their experiences?

 I enjoyed reading this and will recommend this paper to colleagues or students who are considering NGT in a virtual environment.

If evidence from practice is presented, are all the underlying source data available to ensure full reproducibility?

Not applicable

Is the topic of the practical tips discussed accurately in the context of the current literature

Yes

Are all factual statements correct and adequately supported by citations?

Yes

Are the conclusions drawn balanced and justified on the basis of the presented arguments?

Yes

Are arguments sufficiently supported by evidence from the published literature and/or the authors’ practice?

Yes

Reviewer Expertise:

Qualitative research methods

I confirm that I have read this submission and believe that I have an appropriate level of expertise to confirm that it is of an acceptable scientific standard.

# Peer review: Reviewer response for version 1

**DOI:** 10.21956/mep.21000.r33046

**Published:** 2023-04-11

**Authors:** Zarrin Siddiqui

**Affiliations:** 1 College of Health Sciences, VinUniversity, Hanoi, Hanoi, Vietnam

This article intends to provide guidance to those who plan to use Nominal Group Techniques for research and how they can benefit from using virtual platforms. The manuscript is clearly written and uses easy to understand language for novice researchers. 

 There are two suggestions that authors may incorporate in the manuscript.

1. There is a reference to the authors' experience in several tips and the abstract and it also states in abstract and introduction that these tips are based on the experience. I suggest to add a couple of more lines about the 'experience'.
2. One missing section is the ethical considerations in the planning of the virtual NGT and that ethical approval will require more robust measures both from researcher and institution's perspectives. Thank you

If evidence from practice is presented, are all the underlying source data available to ensure full reproducibility?

Not applicable

Is the topic of the practical tips discussed accurately in the context of the current literature

Yes

Are all factual statements correct and adequately supported by citations?

Yes

Are the conclusions drawn balanced and justified on the basis of the presented arguments?

Yes

Are arguments sufficiently supported by evidence from the published literature and/or the authors’ practice?

Yes

Reviewer Expertise:

faculty development, assessment & remediation, mental health and well being.

I confirm that I have read this submission and believe that I have an appropriate level of expertise to confirm that it is of an acceptable scientific standard.
